# *In vitro *effects of relaxin on gene expression in porcine cumulus-oocyte complexes and developing embryos

**DOI:** 10.1186/1477-7827-9-15

**Published:** 2011-01-27

**Authors:** Jean M Feugang, Jonathan M Greene, Scott T Willard, Peter L Ryan

**Affiliations:** 1 Department of Animal & Dairy Sciences, Mississippi State University, 4025 Wise Center Dr, Mississippi State, MS 38762, USA; 2 Department of Biochemistry & Molecular Biology, Mississippi State University, 402 Dorman Hall, Mississippi, MS 38762, USA; 3 Department of Pathobiology & Population Medicine, Mississippi State University, 240 Wise Center Dr, Mississippi State, MS 38762, USA

## Abstract

**Background:**

Relaxin hormone peptide is found in porcine follicular and utero-tubal fluids, but its possible actions during early embryo development are still undetermined. Here, we investigated the effects of porcine relaxin during oocyte maturation and embryo development, and gene expression in the pig.

**Methods:**

Immature cumulus-oocyte complexes (COCs) were obtained from ovarian follicles of sows. In experiment 1, COCs were matured in the presence of 0, 20, or 40 ng relaxin/ml, or 10% (v/v) porcine follicular fluid. In experiment 2, COCs were *in vitro *matured, fertilized and resulting embryos were cultured in the presence of 0, 20, or 40 ng relaxin/ml. In experiment 3, COCs were matured in the presence of 40 ng relaxin/ml, fertilized and zygotes were cultured as indicated in experiment 2. We evaluated the proportions of matured oocytes in experiment 1, cleaved and blastocysts on Day 2 and Day 7 post insemination in all experiments. The total cell number of blastocysts was also evaluated. In parallel, transcription levels of both relaxin and its receptors (RXFP1 and RXFP2), as well as a pro- (Bax) and anti- (Bcl2-like 1) apoptotic-related genes were determined. All data were analyzed by ANOVA and significant differences were fixed for P < 0.05.

**Results:**

In experiment 1, relaxin significantly increased the proportions of matured oocytes and cleaved embryos, as well as the expression level of RXFP2 mRNA compared to RXFP1 (P < 0.05). There was no effect on endogenous expression of relaxin and Bcl2-like1/Bax ratios. In all experiments, relaxin did not affect the proportions of blastocysts, but did significantly increase their total cell numbers (P < 0.05). Furthermore, no effect of relaxin was observed on Bcl2-like1/Bax expression ratios, which were similar between groups.

**Conclusions:**

Exogenous relaxin influences its own receptors expression, improves oocyte nuclear maturation. Its beneficial effect on total cell number of blastocysts appears to be through a Bcl2-like1/Bax-independent mechanism.

## Background

Gene expression is one of the major events occurring during oocyte maturation and early embryo development, and its perturbation in culture system may contribute to the limited production of high quality blastocysts [1-3]. Numerous strategies such as low oxygen tension during culture, co-culture, and utilization of simple or complex media supplemented with various molecules have been developed to improve embryo culture efficiencies [1,4-6]. These molecules such as relaxin are generally found in the vicinity of oocytes and embryos. Relaxin is a peptide hormone found at higher levels in follicular fluids and genital tract secretions of various species [7-11]. Despite its pleiotropic roles reported in various reproductive and non-reproductive tissues [11-13], little is still known on embryonic effects of relaxin during early pregnancy.

In livestock, the pig is the primary animal model used for the study of relaxin effects [11,14,15] due to the important production of relaxin in both pregnant and non-pregnant pigs. Indeed, pigs exhibit high levels of relaxin in ovarian follicular fluids [8,14], and its secretions by corpora lutea and uterine cells significantly increase during the post-ovulatory period [11,14,16-18]. Numerous studies have reported various actions of relaxin on pig reproductive tissues such as cervical and uterine tissues, including modulation of embryo implantation during pregnancy in rats [19-21]. Despite these physiological conditions, it is not known whether relaxin influences oocyte maturation and/or embryo development in the pig.

Relaxin hormone peptide exerts autocrine, endocrine, and paracrine effects through membrane receptors known as RXFP1 and RXFP2 [22-24]. These receptors have been detected in porcine reproductive tissues [25,26], including oocytes and embryos [27]. But, their potential activation by relaxin and further implications on oocyte maturation and embryo development are still unknown.

From this background, the present study aims to bring more insight into the biological functions of relaxin during early pregnancy using pigs as experimental models. We evaluated the effect of relaxin on the proportion of oocytes resuming nuclear maturation and on embryo development, as well as the total cell number of blastocysts. We determined the effects of relaxin on endogenous relaxin, RXFP1, RXFP2, and pro- (Bax) and anti- (Bcl2-like1) apoptotic-related genes expressions in porcine oocytes and *in vitro *produced embryos.

## Methods

Unless otherwise indicated, all chemicals and reagents were purchased from Sigma-Aldrich (Saint Louis, MO, USA) for embryo production or Invitrogen Co. (Carlsberg, CA, USA) for gene expression. Lyophilised relaxin hormone (pRLN) produced from pregnant sow ovaries was a gift from Dr. C Bagnell [15]. It was dissolved in deionized water (10 μg/ml), aliquoted, and stored as stock solution at -80°C. Pre-warmed solutions of NaCl (0.9%; w/v) and Hepes-buffered Tyrode Lactate solution (Vigro-Bioniche, Pullman, WA, USA) supplemented with polyvinyl alcohol (PVA: 0.1%; w/v) and pyruvate (100 μM) were used to wash the ovaries, and the oocytes and the embryos, respectively. Oocytes were matured in TCM199+L-glutamine medium supplemented with PVA (1%; w/v), glucose (2.8 mM), pyruvate (0.91 mM), cysteamine (0.57 mM), EGF (10 ng/ml), and FSH (0.4 μg/ml) and fertilized in the modified Tris-buffered medium (mTBM) containing caffeine (2 mM) and BSA-fraction V (0.1%; w/v). Embryos were cultured in NCSU-23 medium (Millipore, Billerica, MA, USA) supplemented with BSA-FAF (0.4%; w/v). All media contained 10 μl/ml antibiotics (gentamycin and penicillin G/Streptomycin sulphate) and were pre-incubated for at least 2 h before use. Oocytes and embryos were placed in 4-well dishes (Nunc, Roskilde, Denmark) for incubations under a humidified atmosphere of 39°C and 5% CO_2 _in air.

### Cumulus-oocyte complex collection, in vitro maturation (IVM), fertilization (IVF) and embryo culture (IVC)

Sow (Yorkshire-Landrace) ovaries were harvested at a local abattoir (Southern Quality Meats, Pontotoc, MS, USA) and washed in the laboratory. Immature cumulus-oocyte complexes (COCs) were aspirated from follicles of 3-6 mm diameter. Oocytes with homogenous ooplasm and surrounded by cumulus cells were selected for maturation per groups of 50-75 COCs in 500 μl of medium. After 44 h incubation, COCs were collected and transferred into 250 μl of fertilization medium. High motile spermatozoa of pooled semen (Prestage Farms, West-Point, MS, USA) were purified through a discontinuous percoll gradient (90%-45%) and 250 μl of suspended sperm cells (12 × 10^5 ^spermatozoa/ml) was used to fertilize the COCs. Both gametes were co-incubated (Day 0 post-insemination or Day 0pi). Presumptive zygotes were harvested at 18 hpi and mechanically separated from the cumulus cells followed by the transfer of cumulus free-zygote into drops of cultured medium (1 embryo/1-2 μl) overlaid with mineral oil. Embryos were incubated for up to 6 days (Day 7pi).

### Experiment 1: Developmental and gene expression effects of pRLN added during IVM

Cumulus-oocyte complexes were matured in the presence of 0, 20 or 40 ng/ml pRLN, or 10% (v/v) pFF. They were subsequently fertilized, and the presumptive zygotes were cultured until the blastocyst stage. All steps were processed as described above.

In a preliminary study aimed at evaluating our culture conditions, a total of 550 COCs (in 4 independent replicates) were matured in the presence of 10% pFF. Proportions of 44 ± 4% (mean ± SD) cleaved embryos and 17 ± 3% (mean ± SD) blastocysts (per cleaved: 40/229) were obtained.

#### Evaluation of oocyte maturation

COCs were collected after maturation (44 h) followed by a mechanical separation of oocytes and cumulus cells. Denuded oocytes were fixed in acetic acid: ethanol mix (1:3, v/v; 24 h at 4°C), stained with 1% aceto-orcein^, ^and mounted onto slides for nuclear status evaluation under a phase-contrast microscope (Nikon Instruments Inc.; Melville, NY). Oocytes with germinal vesicle, metaphase I, or metaphase II nucleus features were classified as immature (GV), resuming meiosis (MI), or mature (MII) oocytes.

#### Evaluation of embryo development

Proportions of cleaved and blastocyst stage embryos were evaluated on Day 2pi and Day 7pi, respectively.

#### Sample collection for gene expression

Groups of 30 immature and 30 mature COCs were mechanically separated from their respective cumulus cells under a stereomicroscope. Isolated cumulus cells and cumulus free (or denuded) oocytes and other groups of 10 cleaved embryos (2-4 cells) and 3 blastocysts (Day 7pi) were separately selected under microscope based on morphological criteria (homogenous cytoplasm) for the gene expression study. All samples were stored at -80°C until analysis.

### Experiment 2: Developmental and gene expression effects of pRLN added during IVM and IVC

Cumulus-oocyte complexes were maturated in the presence of 0, 20 or 40 ng pRLN/ml followed by their fertilization. Resulting zygotes were cultured for up to 6 days (Day 7pi) in the presence of 0, 20 or 40 ng/ml of pRLN. All steps were processed as described above and embryo production was done in duplicate for developmental and gene expression studies.

#### Developmental evaluation of relaxin effects

Cleaved and blastocyst stage embryos were recorded on Day 2pi and Day 7pi, respectively. Day 7pi-blastocysts were placed on microscope slides, air dried, fixed (for 24 h in absolute alcohol), and stained with the Hoechst 33342 dye. Blastocyst cell numbers were evaluated under an Epifluorescence microscope (Nikon Inc., Melville, NY, USA).

#### Sample collection for gene expression

Groups of 10 zygotes, 10 cleaved (2-4 cells) and 3 blastocyst stage embryos were selected based on their morphology under a stereomicroscope and stored per groups at -80°C until analysis.

### RNA isolation and RT-PCR

Total RNA were isolated from frozen-thawed samples (RNeasy Micro Kit, Qiagen Inc., Valencia, USA), reverse-transcribed into cDNA (Superscription III Platinum^® ^Two-Step qRT-PCR Kit) which were used for detecting the expression levels of relaxin and its receptors (RXFP1 and 2) and apoptotic related genes (Bax and Bcl2-like1). Expression levels of β-actin in each biological sample were determined for normalization. To this end, semi-quantitative (Taq DNA polymerase kit) and real-time (SYBR^® ^GreenER q PCR SuperMixes for iCycler) PCR techniques were employed. Table 1 summarizes the primer pairs' characteristics. The PCR conditions were as followed: 5 min at 95°C; 45 cycles of [30 sec at 95°C, 30 sec at the optimal annealing temperature (Table 1) and 30 sec at 72°C]; 10 min at 72°C. Real time PCRs using tested (Bax, Bcl2-like1, Relaxin, RXFP1 and RXFP2) and internal control (β-actin) genes' primer pairs were performed, and the comparative Ct method was used to determine the transcript levels as previously described [28]. Relaxin, RXFP1 and RXFP2 gene expression data are indicated as relative expression to β-actin, while individually normalized Bax and Bcl2-like1 to β-actin are shown as Bcl2-like1/Bax ratios. All PCR products were further resolved on 1.5% agarose gels to verify of the amplification quality given the size of the amplicon products.

**Table 1:** Primer sequence characteristics

Gene symbols	GenBank Acc.#, (NCBI)	Primer sequences (5'-3')		AT ** (**^**o**^**C)**	Amplicon sizes (bp)
RXFP1	CA994862.1	S: AS:	AGGCTGACGAGGACAACT CAGAACCGACCAAGCATT	52.5	132
RXFP2	CA997681.1 (XM_001927967.1)	S: AS:	CATCTGCTGGATTCCCGTAT TTCAAGGCACTGTTCACC	55	117
RELAXIN	NM213872.1	S: AS:	TGTGGCTCCGTCTCCTGGGG GTTGCCTTCAGCTCCTGTGGC	55	164
BCL2-like1	NM214285.1	S: AS:	TGAATCAGAAGCGGAAACCC GCTCTAGGTGGTCATTCAGGTAAG	60	416
BAX	AJ606301	S: AS:	TTTCTGACGGCAACTTCAACTG AGCCACAAAGATGGTCACTGTCT	60	238
β-ACTIN	U07786	S: AS:	ACTGGCATTGTCATGGACTCTG AGTTGAAGGTGGTCTCGTGGAT	60	397

S, sens; AS, AntiSens

In parallel, sections of electrophoresis gels containing relaxin, RXFP1 and RXFP2 PCR products generated from porcine neonates uterine cells (Ut) and ovarian corpora lutea (CL) samples were excised to test their authenticities. Agarose gel sections were purified and submitted for sequencing (BigDye terminator V1.1 cycle sequencing kit; Applied Biosciences Inc., Foster City, USA) followed by their BLAST on pig genome (NCBI repository database). Bax, Bcl2-like1 and β-actin primer sequences published by Wang et al. were used in this study [28].

### Statistical analysis

All experiments were repeated at least three times and RNA samples obtained from each experimental replicate. One-way ANOVA (SYSTAT, Systat software Inc., Chicago, IL, USA) followed by the Fisher's Least Square Difference test for pairwise comparisons were used to analyze the pRLN effects. The Student's t-test was used to compare the expression levels of Bax, Bcl2-like1, RXFP1, and RXFP2 mRNA within the sample type (MCC: mature cumulus cell, or MII: mature oocyte). Results are expressed as mean (± SD) for gene expression or (± SEM) for developmental data, and P < 0.05 are fixed for significant differences.

## Results

### Experiment 1: Development and gene expression effects of pRLN added IVM

#### Developmental effects

The presence of pRLN during IVM did not affect the cumulus cell expansion (data not shown); however, it did significantly increase the proportion of oocytes that resumed meiosis (79% ± 4%, 87% ± 3%, and 91% ± 3%, for 0, 20 and 40 ng pRLN/ml, respectively, P < 0.05), and, subsequently, increased the proportions of oocytes that reached metaphase II (68% ± 5%, 80% ± 4% or 88% ± 4% for 0, 20 or 40 ng pRLN/ml, respectively, P < 0.05; ANOVA; Table 2).

**Table 2:** Effect of relaxin on porcine oocyte maturation

pRLN during IVM (ng/ml)	Total Oocytes (N)	Nuclear maturation status of oocytes		
		GV% (n)	MI% (n)	MII% (n)
0	451	21 ± 4 (93)^a^	11 ± 5 (51)^a^	68 ± 5 (307)^a^
20	472	13 ± 3 (61)^ab^	7 ± 2 (31)^a^	80 ± 4 (380)^b^
40	331	9 ± 3 (30)^b^	3 ± 3 (9)^b^	88 ± 4 (291)^c^
P values (ANOVA)		10^-3^	0.03	10^-4^

^abc ^Different superscripts within the same column indicate significant difference (P < 0.05; ANOVA). Data are mean values (± SEM) of at least 4 independent replicates.

Furthermore, we evaluated the effect of relaxin on embryo development by maturing a total of 1,169 COCs in the presence of 0, 20 and 40 ng pRLN/ml (4 to 7 independent replicates, Table 3). Only 40 ng pRLN/ml significantly increased the cleavage and blastocyst rates in comparison to the control group (51 ± 5% and 10 ± 3% vs. 37 ± 4% and 12 ± 3%, respectively; P < 0.05). There were no significant differences between the 20 ng pRLN/ml treatment and the control. Moreover, the mean cell number of blastocysts was significantly higher in the 40 ng pRLN/ml group (38 ± 3) compared to others (control: 31 ± 4 and 20 ng pRLN/ml: 32 ± 6; P < 0.05), which appeared similar.

**Table 3:** Developmental effects of relaxin added during oocyte maturation

pRLN during IVM (ng/ml)	Total Zygotes (N)	% of cleaved at Day 2pi (n)	Blastocyst formation at Day 7pi		
			Total (T)	% (T/N)	Cell number (n)
0	383	37 ± 4 (141)^a^	45	12 ± 3^a^	31 ± 4 (16)^a^
20	344	40 ± 4 (135)^a^	23	8 ± 4^a^	32 ± 6 (12)^a^
40	442	51 ± 5 (226)^b^	46	10 ± 3^a^	38 ± 3 (12)^b^

^ab ^Different superscripts within the same column indicate significant difference (P < 0.05; ANOVA). Data are mean values (± SEM) of at least 5 independent replicates.

#### Gene expression effects

**Figure 1:**
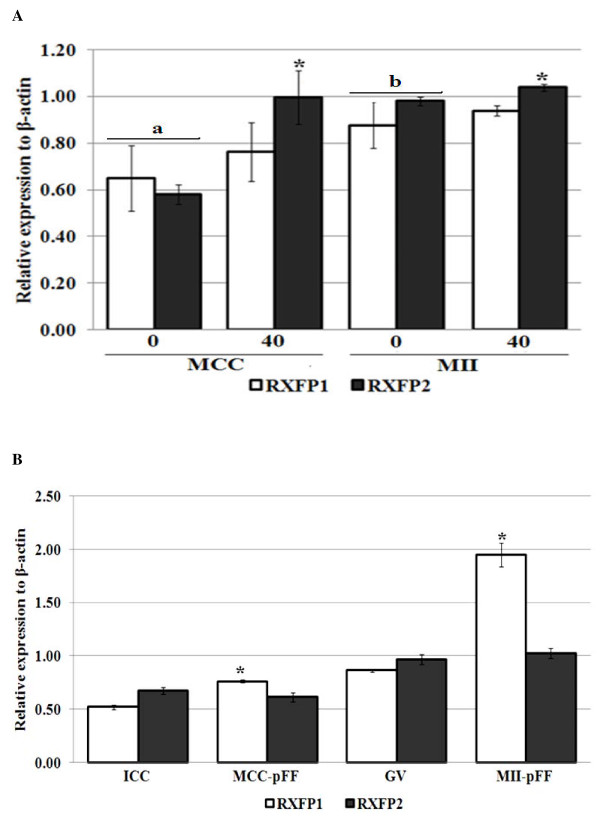
**Effect of porcine relaxin (pRLN) and follicular fluid (pFF) on RXFP1 and RXFP2 gene expression in *in vitro *matured oocyte porcine**. Figures represent real-time PCR results of RXFP1 and RXFP2 gene expression in oocytes matured in teh presence of 0 or 40 ng/ml pRLN (**A**), and 0 or 10% (v/v) pFF (**B**). Data are relative expression to their corresponding internal control, β-actin; and are shown as mean ± SD of at least three independent replications. Letters (a,b) represent significant differences between immature oocytes and cumulus for both genes, while asterisks (*) indicate significant differences between treated groups and their corresponding controls within the same gene (P < 0.05, ANOVA). Immature oocyte and cumulus cells are labelled as GV and ICC, while those that are matured in the presence of pRLN are indicated as MII-40 and MCC-40, or MII-pFF and MCC-pFF for those matured in the presence of pFF.

**Figure 2:**
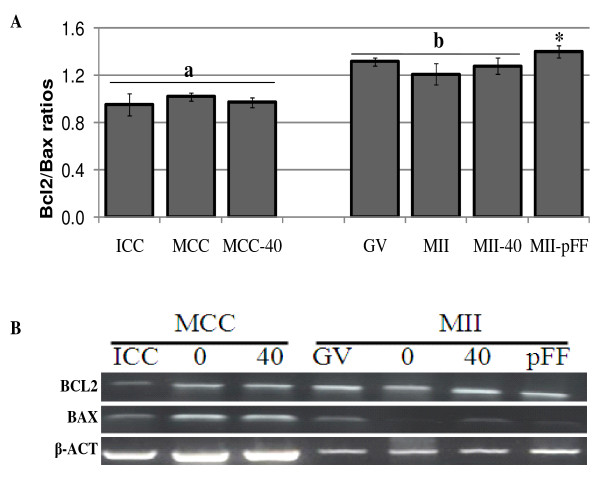
**Effect of porcine relaxin (pRLN) and follicular fluid (pFF) on pro- (Bax) and anti-(Bcl2-like 1) apoptotic genes expression in oocytes and cumulus cells**. Total RNA were extracted from groups of 30 oocytes and corresponding cumulus cells before and after *in vitro *maturation in the presence of 0 or 40 ng pRLN/ml, or 10% (v/v) pFF. Gene expression was assessed using real-time PCR (**A**) followed by agarose gel electrophoresis of resulted PCR products (**B**). Analyses were performed in three independent replicates and data were expressed as relative to their corresponding internal control, β-actin. The figure represents the ratio (mean ± SD) of individually normalized Bcl2-like1/Bax gene expression. At all stages, oocytes contained significantly higher values than cumulus cells (a,b; P < 0.05, ANOVA), and pRLN had no effects on Bcl2-like1/Bax ratios (MII-40 and MCC-40), while pFF significantly increased this ratio in oocytes compared to other groups (*; P < 0.05, ANOVA). Immature cumulus cells and oocytes are indicated by ICC and GV, respectively. Cumulus-oocyte complexes matured in absence of pRLN are indicated by MCC and MII, corresponding to mature cumulus cells and oocytes. Cumulus cells and oocytes matured in the presence of pRLN are indicated by MCC-40 and MII-40, respectively.

**Figure 3:**
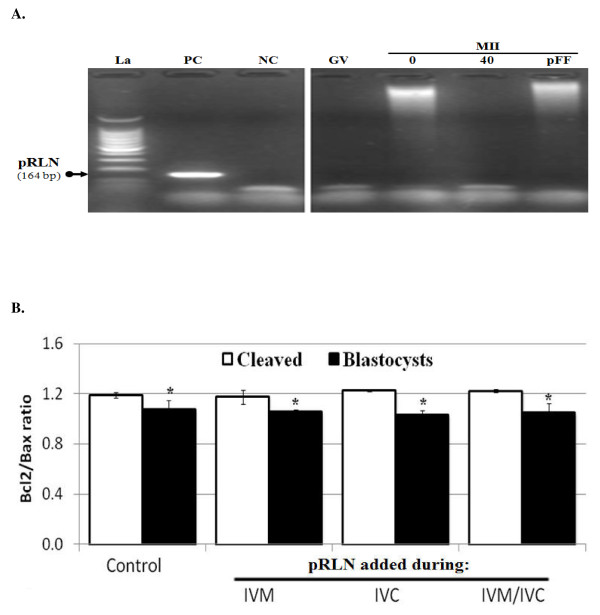
**Effect of porcine relaxin (pRLN) on relaxin, pro- (Bax) and anti- (Bcl2-like 1) apoptotic genes expression in porcine embryos**. Figure represent representative agarose gel of relaxin amplification through end-point PCR (**A**) and real-time PCR results of Bax and Bcl2-like1 amplifications (**B**). COCs were collected immature (GV) and matured in the presence of 0 (0), 40 ng pRLN/ml (40) or 10% pFF (pFF). Cleavage embryos and blastocysts were obtained from various experiments: exposure to 40 ng pRLN/ml during oocyte (IVM) or embryo culture (IVC), or during both periods (IVM/IVC). The control group corresponds to embryos produced without any exposure to pRLN. Embryos were analyzed per groups in three independent replicates. Data were expressed as relative to their corresponding internal control, β-actin, and shown as ratios (mean ± SD) of individually normalized Bcl2-like1/Bax gene expression. There was no effect of pRLN compared to the control group, and values in blastocysts were always significantly lower than that of cleaved embryos within the same treatment group (*; P < 0.05, ANOVA). Neonate porcine uterine RNA and water were used as positive control (PC) and negative control (NC), respectively.

We evaluated the effect of relaxin on Bax, Bcl2-like1, relaxin, RXFP1 and RXFP2 gene expression in both cumulus cells and oocytes. With exception of relaxin, all other gene transcripts were detected in both cell types (Figure 1, 2 and 3). Their expression levels in cumulus cells were always lower than that in oocytes (P < 0.05). The concentration of 40 ng pRLN/ml significantly increased RXFP2 mRNA transcript levels in oocytes and cumulus cells (Figure 1A; P < 0.05), but had no effect on Bcl2-like1/Bax ratios in both cell types (Figure 2). The presence of 10% porcine follicular fluid (pFF) significantly increased RXFP1 mRNA amounts in mature cumulus cells and oocytes, as well as the Bcl2-like1/Bax ratio in mature oocytes (Figure 1B and 2; P < 0.05). However, relaxin transcripts were not detected in oocytes, and neither pRLN nor pFF were able to induce its de novo expression (Figure 3A).

### Experiment 2: Developmental and gene expression effects of pRLN added during IVM and IVC

#### Developmental effects

A preliminary study was conducted with a total of 1,343 zygotes cultured in culture media containing 0 (control), 20, or 40 ng pRLN/ml. We found no significant effects of relaxin on the proportions of cleaved embryos and blastocysts. However, the mean cell numbers of blastocysts were significantly increased in the presence of pRLN (control: 31 ± 4, 20 ng/ml: 49 ± 12, 40 ng/ml: 38 ± 8; P < 0.05, ANOVA). Based on these results, we evaluated the effect of pRLN added during oocyte maturation (0 or 40 ng/ml) and embryo culture (0, 20, or 40 ng/ml).

Oocytes matured in the presence of 40 ng pRLN/ml followed the culture of resulting zygotes in the presence of 20 or 40 ng pRLN/ml (Treated-1 and Treated-2, respectively). Embryos produced without any exposure to pRLN served as the control group. A total of 949 were used in at least three independent replicates to evaluate the effect of pRLN added during both maturation (Treated-1) and embryo culture (Treated-2). Results summarized in Table 4 revealed a negative effect of pRLN on the cleavage rate in group Treated-1 (40-20) compared to Treated-2 (40-40) and control (33 ± 5% vs. 51 ± 8% and 48 ± 3%, respectively; P < 0.05), whereas no differences in term of blastocysts rates were observed. However, the mean cell number of blastocysts was significantly increased in the Treated-2 group (43 ± 6) compared to the control group (31 ± 3; P < 0.05).

**Table 4:** Effects of relaxin added during both oocyte maturation and embryo culture

pRLN (ng/ml) during		Total Zygotes (N)	% of cleaved at Day 2pi (n)	Blastocyst formation at Day 7pi		
IVM	IVC			Total (T)	% (T/N)	Cell number (n)
0	0	324	48 ± 3 (156)^a^	24	8 ± 2^a^	31 ± 3 (24)^a^
40	20	272	33 ± 5 (91)^b^	19	7 ± 2^a^	33 ± 3 (12)^a^
40	40	353	51 ± 8 (172)^a^	33	10 ± 2^a^	43 ± 6 (10)^b^

^abc ^Different superscripts within the same columns indicate significant difference (P < 0.05; ANOVA). Data are mean values (± SEM) of at least 5 independent replicates.

#### Gene expression effects

We evaluated the effect of 40 ng pRLN/ml added during oocyte maturation (IVM), embryo culture (IVC), or both periods (IVM/IVC) on Bax and Bcl2-like1 gene expression in cleaved and blastocyst stage embryos. Figures 3B and 3C show that the Bcl2-like1/Bax ratios were significantly higher in cleaved embryos compared to blastocysts (P < 0.05, t-test). However, the moment of relaxin exposure (IVM or IVC) had no significant effect on these ratios (ANOVA).

## Discussion

Numerous physiological roles have been attributed to relaxin [10,11]; but its effects during embryo development are still unknown despite its presence in gamete and embryo environments. Here, we could not detect the expression of relaxin in porcine COCs but found an accumulation of its receptors RXFP1 and RXFP2 mRNA in COCs which appeared to be modulated by exogenous relaxin. In addition, relaxin moderately influenced embryo development but significantly increased the total cell number of blastocysts irrespective of the moment of its addition in the media (oocyte maturation and/or embryo culture).

The present study reports developmental effects of relaxin used at concentrations similar to those found in the literature [29-31]. Indeed, variable but large amounts of relaxin are found in follicular fluids surrounding porcine oocytes, while pre-implantation embryos are suspected to be exposed to unknown amounts of relaxin during their utero-tubal progression and development (corresponding to the ovarian luteal phase). Beneficial roles of relaxin have been demonstrated during follicle growth and ovulation [7,14], uterine receptivity and activity process in various species such as pigs [14], primates [32,33] and rodents [21,34,35]. To our knowledge, the current study is the first to report developmental effects of exogenous relaxin during both oocyte maturation and early embryo development.

These effects are results of putative interactions between relaxin and its receptors located on plasma membranes of target cells [22,24,36]. In previous studies, we reported the presence of these receptors on porcine oocytes and embryos [27]. Here, we found that the addition of porcine relaxin in a follicular fluid-free maturation medium induces an accumulation of RXFP2 mRNA in COCs. This effect is surprising given that relaxin is the specific ligand of RXFP1, although it can bind with low affinity to RXFP2. This observation suggests a possible role of RXFP2 receptors during mammalian oocyte maturation process in the pig, as also suggested in rhesus macaque [37] and rat [38] oocytes expressing RXFP2 mRNA. In-depth studies of INSL3-RXFP2 complex during oocyte maturation and embryo development will definitely contribute to better characterize the full developmental effects of this complex.

Nevertheless, given the complexity of molecular interactions existing within the follicular fluid, a likely activation of RXFP1 receptors during *in vivo *oocyte maturation cannot be ruled out; and seems to be highlighted in the current study by the significant increase of RXFP1 mRNA in oocytes matured in the presence of porcine follicular fluid alone. The existence of such interactions may also explain our contradictory results with a recent study reporting a lack of relaxin effect on nuclear maturation of pig oocytes [39]. These authors tested the effect of recombinant human relaxin added in maturation medium already containing porcine follicular fluid (10%) and whose high relaxin contents [8,40] and beneficial effect on oocyte maturation [41,42] could have interfered with their results. In our study, oocytes were matured in the absence of follicular fluid, and the supplementation of the basal medium with relaxin significantly increased the proportions of oocytes resuming and accomplishing meiotic maturation. This beneficial effect of relaxin could be explained by its interactions with its receptors leading to an inhibition of oocyte meiosis blocking factors such as cyclic adenosine monophosphate (cAMP) as previously suggested [38].

Most interesting, the developmental effects of relaxin suggest both direct and indirect effects on embryos and oocytes, respectively. While embryos were freed of the surrounding cumulus cells after fertilization, oocytes were matured together with their surrounding cumulus cells. Using rodent oocytes, Kawamura et al. have proposed an indirect effect of relaxin on its targets in a paracrine way [38]. Irrespective of the moment of its addition, the increased total cell number of blastocysts appears as the hallmark of relaxin during early embryo development. Indeed, beneficial effects of relaxin were limited at the cleavage stage (high proportions) following oocyte maturation in the presence of relaxin, while any effects were observed on blastocyst formation compared to the control groups.

Developmental effects of relaxin in our study indicate possible perturbations of molecular mechanisms associated with cell cycle, and specifically cell proliferation. A recent study has demonstrated a positive effect of relaxin on glutathione (GSH) synthesis in *in vitro *matured porcine oocytes [39]. Indeed, GSH is a routine indicator or oocyte cytoplasmic maturation [43], whose beneficial effects on blastocyst formation and quality (hatching and cell number) are well-documented [44-48]. Therefore, we suggest that the increase of blastocyst cell number in our study is probably associated to an increase of GSH in oocytes and/or developing embryos.

In addition, production of GSH induces an imbalance of reduced-oxidative (REDOX) status of cells leading to potential modifications of cellular functions such as cell cycle (incidence of apoptosis) [49]. Therefore, we anticipated an activation of apoptotic-related genes by relaxin. Apoptosis is an important biological process during blastocyst formation of which fine-tuned balances between pro-apoptotic (Bax) and anti-apoptotic (Bcl2-like1) genes are critical for the survival of embryos [50]. Our study revealed that Bcl2-like1/Bax ratios were always in direction of Bcl2-like1 transcripts which is favorable to embryo survival. The presence of relaxin during culture did not significantly affect this ratio which remained comparable between groups.

## Conclusions

In summary, our findings suggest that the increase in the total cell number in blastocysts is the hallmark effect of exogenous relaxin. Given that the cell number of blastocysts is an important gauge for *in vitro*-produced embryos, the supplementation of porcine embryo culture media with relaxin could be considered. However, the effects of relaxin on the expression of developmentally important genes should be investigated.

## Competing interests

The authors declare that they have no competing interests.

## Authors' contributions

JMF conceived and performed all the experiments, and wrote the first draft of the manuscript. JMG contributed to the in vitro embryo production. STW and PLR contributed to the design of the study, revised and approved the manuscript. All authors read and approved the final manuscript.
